# Neonatal jaundice in Ghanaian children: Assessing maternal knowledge, attitude, and perceptions

**DOI:** 10.1371/journal.pone.0264694

**Published:** 2022-03-03

**Authors:** Nana Ayegua Hagan Seneadza, Genevieve Insaidoo, Hilda Boye, Mary Ani-Amponsah, Terence Leung, Judith Meek, Christabel Enweronu-Laryea

**Affiliations:** 1 Department of Community Health, University of Ghana Medical School, College of Health Sciences, University of Ghana, Accra, Ghana; 2 Department of Child Health, Holy Family Hospital, Nkawkaw, Eastern Region, Ghana; 3 Department of Child Health, Korle Bu Teaching Hospital, Accra, Ghana; 4 School of Nursing and Midwifery, College of Health Sciences, University of Ghana, Accra, Ghana; 5 Department of Medical Physics & Biomedical Engineering, Malet Place Engineering Building, University College London, London, United Kingdom; 6 Neonatal Care Unit, EGA Wing, University College London Hospitals, London, United Kingdom; 7 Department of Child Health, University of Ghana Medical School, College of Health Sciences, University of Ghana, Accra, Ghana; Monroe Carell Junior Children’s Hospital at Vanderbilt, UNITED STATES

## Abstract

**Background:**

Neonatal jaundice (NNJ) is a major cause of preventable childhood mortality and long-term impairment especially in countries with significant prevalence of the inherited condition, glucose-6-phosphate dehydrogenase (G6PD) defect. In Ghana, routine screening of pregnant women for G6PD defect is standard care. Prevention of poor health outcomes from NNJ is contingent on population health literacy and early diagnosis. As part of a project to evaluate a screening tool for NNJ, we assessed the knowledge, attitude, and perceptions of Ghanaian mothers on NNJ at baseline.

**Methods:**

Using a cross-sectional design, mothers attending antenatal and postnatal clinics at 3 selected health facilities in 2 geographical regions of Ghana were interviewed. Data on mothers’ understanding, perceptions, beliefs, and actions towards NNJ were evaluated. Chi-square test was used to determine the association between selected maternal characteristics and knowledge, attitude, and perception to NNJ.

**Results:**

Of the 504 mothers interviewed, 428(85.4%) had heard about NNJ, 346 (68.7%) said the earliest signs are seen in the eyes, 384(76.2%) knew NNJ may be harmful and 467(92.7%) recommended seeking healthcare for the jaundiced newborn. None of the women knew about G6PD or their G6PD status following antenatal screening. Most did not know the signs/symptoms of severe NNJ. Of the 15 mothers who had had a jaundiced neonate, cost was the most perceived (8 out of 15) barrier to accessing health care. There were significant associations (p-value ≤ 0.05) between maternal age, educational level, and knowledge of NNJ.

**Conclusion:**

Despite the high level of awareness of NNJ, gaps still exit in the knowledge, attitudes and perceptions of mothers concerning NNJ. Improving education of women about the causes, symptoms/signs, and the role of G6PD in severe NNJ is recommended. Addressing barriers to accessing healthcare for the jaundiced infant may enhance timely management of NNJ and reduce the associated complications and mortality.

## Introduction

By the third day after birth, up to 60% of term and 80% of preterm newborn infants have “yellow” pigmentation of the eyes and skin due to neonatal jaundice (NNJ) [1]. In most infants, NNJ is physiological and resolves spontaneously by the end of the first week (term) or second week (preterm) after birth. Nevertheless, a significant number of infants have an associated pathological cause of jaundice and will need treatment, mostly with phototherapy or exchange blood transfusion for severely affected infants [2]. Globally, NNJ is among the top 5 causes of hospital readmission of newborn infants [3]. In severe NNJ, unconjugated hyperbilirubinaemia causes significant brain damage (kernicterus), death, hearing loss, cerebral palsy and other impairments, especially in low-resource settings [4–6]. Severe NNJ is the major and most preventable cause of cerebral palsy in children attending a tertiary neurology clinic in Ghana [7], and the burden has not significantly improved in the West African region in the last five decades [8, 9].

The causes and risk factors for NNJ include blood group incompatibilities, birth injury, metabolic and endocrine disorders, and inherited conditions such as glucose-6-phosphate dehydrogenase (G6PD) deficiency, which is prevalent in sub-Saharan Africa [10]. In Ghana, antenatal screening for G6PD deficiency is standard care, most women receive antenatal care and skilled care at birth has improved over time [11]. In many low-resource settings, mothers with a normal birth at health facilities are discharged before the usual manifestation of NNJ, and early detection and care-seeking primarily depends on the mother’s knowledge and attitude towards NNJ [12–14]. Several studies in low-resource settings indicate that the level of antenatal care coverage and institutional delivery did not guarantee optimal knowledge and practice of mothers regarding NNJ [12, 15–19]. In keeping with a holistic approach to implementing new health technologies, we explored the knowledge, perceptions, beliefs, approach, and actions of mothers towards NNJ at baseline as part of a project evaluating the application of a smartphone-based screening tool for neonatal jaundice. We hypothesized that Ghanaian women accessing antenatal and postnatal services had inadequate knowledge and inappropriate attitudes and perceptions towards NNJ. We also hypothesized that majority of the mothers would know their G6PD deficiency status and the associated risk of NNJ if the mother was G6PD deficient.

## Materials and methods

Using a cross-sectional design, women accessing antenatal and postnatal maternal newborn services in the Greater Accra and Eastern Regions of Ghana were recruited from purposefully selected referral and district level health facilities during August to November 2018. Three health facilities (Greater Accra Regional Hospital, Mamprobi Polyclinic-Accra, and Holy Family Hospital-Nkawkaw) were selected based on the number of births, services provided and location to enable recruitment of women in rural (Holy Family hospital), urban poor (Mamprobi Polyclinic) and urban (Greater Accra Regional Hospital) settings with varying socioeconomic status. The regional referral hospital attends to about 10,000 births annually and each of the other health facilities attend to about 4,000 births annually. Ghana has provided free maternal health services since 2008;maternal services in rural and urban settings have significantly improved over time with 98% of women attending any antenatal visit, 89% attending 4 or more antenatal visits, 79% delivering at a health facility and 81% of newborns receiving postnatal care within 2 days after birth [20]. The study was approved by the Ghana Health Service Ethics Review Committee (GHS-ERC: 014/07/18). Written permission was obtained from managers of the selected health facilities. All women who volunteered to participate provided written informed consent before being recruited into the study.

Females, 18 years or older were eligible if they met any of the following criteria: (a) pregnant with gestational age of 26 weeks or higher and attended antenatal clinic at least twice; (b) on the obstetric ward during day 0–2 after delivery of a live-born infant; and (c) attending the 2-week postnatal clinic at the study site with a live-born infant of any gestational age. At the clinic or on the ward, the nurse in charge gave permission for the interviewers to inform mothers about the study. Women who volunteered to participate were recruited. At each facility, 56 women were recruited from each of three groups described above (168 participants per facility). Recruitment was done consecutively until the sample size was achieved. Sample size was calculated based on the standard formula for cross-sectional studies and similar work done in Southwest Nigeria [19]. Mothers who are healthcare workers of any category were excluded.

Interviewer-administered pre-tested questionnaires were used to collect data on the three thematic areas (knowledge, attitude, and practice) and mothers’ experiences with the health system. Two interviewers administered the questionnaire to each participant in the language of their choice. The interviewers were fluent in the 3 local languages (Twi, Ga, Fanti) commonly spoken at the study sites. Knowledge was assessed based on the mother’s understanding of what NNJ was, when it usually occurred, the causes, symptoms and signs, immediate and long-term effects, and prevention of severe jaundice. Knowledge about maternal G6PD status and how it relates to NNJ were also assessed. Attitude was assessed by determining what mothers thought needed to be done for the jaundiced newborns and whether or not the babies should be assessed in a health facility. Perceptions about how NNJ may be treated including health facility care was assessed.

### Data analysis

The proportions for each variable were determined. For questions which allowed multiple responses, each response was analyzed separately. Knowledge was categorized as adequate when only correct answers were selected by the participants and inadequate when only incorrect responses, a combination of correct and incorrect responses/option ‘don’t know’ were selected [14]. Attitude and perception were categorized as appropriate when only correct answers were selected by the participants and inappropriate when only incorrect responses, a combination of correct and incorrect responses/option ‘don’t know’ were selected. Chi-square test of association was used to determine the association between selected maternal characteristics and their knowledge, attitude, and perception. For this section of the analysis, knowledge was assessed using general knowledge about neonatal jaundice and knowledge of signs and prevention of severe disease. Attitude and perceptions were categorized based on the variables described above. The data were analyzed using IBM Statistical Package for the Social Sciences software (version 20.0).

## Results

A total of 504 mothers were interviewed. The mean age was 28.4(± 6.2) years with majority (87.4%) aged between 20 and 39 years. The four major ethnic groups in Ghana were proportionately represented. Most, 468(92.9%), had been pregnant at least once and 453(89.9%) had at least one live birth. Majority (80.9%) of those who provided information about their work were service and sales workers and were self-employed (72.5%). Less than half (37.9%) had higher than Junior High school education. Of the 15(3%) mothers who had their own child diagnosed with NNJ within 7 days of life, 12 reported that the diagnosis was made by a health worker. Participants’ profile is summarized in Table 1.

**Table 1:** Profile of mothers from antenatal clinics and postnatal wards in Greater Accra and Eastern Regions of Ghana (N = 504).

Characteristics	Frequency (percent)
**Age at last birthday (years)**	
≤ 19	38 (7.5)
20–29	247 (49.0)
30–39	193 (38.3)
40–49	24 (4.8)
No response	2 (0.4)
**Occupation**	
None	43 (8.5)
Professional	28 (5.6)
Service and sales workers	368 (73.0)
Others	16 (3.2)
No response	49 (9.7)
**Ethnicity**	
Ga/Ga Adamgbe	102 (20.2)
Ewe	56 (11.1)
Akan	278 (55.2)
Northern tribes	56 (11.1)
Non-Ghanaian	3 (0.6)
No response	9 (1.8)
**Marital status**	
Never married	20 (4.0)
Cohabiting	178 (35.3)
Currently married	298 (60.1)
No response	8 (1.6)
**Highest level of education**	
No formal or less than primary school completed	22 (4.4)
Primary school completed	82 (16.3)
Junior high school completed	206 (40.9)
Senior high school/ vocational school completed	131 (26.0)
College/University completed	60 (11.9)
No response	3 (4.4)
**Employment status**	
Government/Non-government employee	53 (10.5)
Self-employed	364 (72.2)
Student	14 (2.8)
No formal employment	71 (14.1)
No response	2 (0.4)
**Has had a baby who developed jaundice**	15 (2.9)

### Knowledge about onset, causes and signs/symptoms

Majority, 428(84.9%), had heard about NNJ from health workers (30.6%) or the media (28.8%). When asked about their understanding of NNJ, the commonest response, 339(67.3%), was yellowing of the eyes. Though 384(76.2%) mothers knew NNJ may be harmful to babies, most did not know whether NNJ commonly occurred in the first week of life or was abnormal when it occurred on the first day and after the second week. Eating foods like palm oil was the commonest response given, 201(39.9%), as the cause of NNJ, with prematurity and infections ranking second and third respectively. Spiritual forces were stated as being responsible for NNJ by 11.3% of participants. Overall, 346(68.7%) mothers described the eyes as the part of the body where the first sign of NNJ could be seen and 271(53.8%) felt that a baby not feeding well was an indication of a serious illness. A summary of the findings is shown in Table 2.

**Table 2:** Knowledge of the mothers about the timing, causes and signs/symptoms of neonatal jaundice (N = 504).

Characteristic	Yes [frequency (%)]	No [frequency (%)]	Don’t know [frequency (%)]
**Understanding of neonatal jaundice** *			
Yellowing of eyes	339(67.3)	-	163(32.3)
Yellowing of skin	68(13.5)	-	433(85.9)
Passing deep yellow urine	44(8.7)	-	456(90.5)
Reddening of eyes	1(0.2)	1(0.2)	497(98.6)
Poor feeding	6(1.2)	1(0.2)	492(97.6)
**General knowledge about NNJ** *			
Commonly occurs in first week of life	146(29.0)	56(11.1)	302(59.9)
Normal in most babies in the first week	50(9.9)	95(18.8)	359(71.2)
Normal when it occurs on the first day	48(9.5)	95(18.8)	361(71.6)
Normal if seen after the second week	44(8.7)	95(18.8)	365(72.4)
May be harmful to some babies	384(76.2)	3(0.6)	117(23.2)
May be associated with poor feeding	100(19.8)	34(6.7)	369(73.2)
May cause abnormal movement/seizure	132(26.2)	27(5.4)	344(68.3)
**Knowledge about the causes of NNJ** *			
Infections	117(23.2)	71(14.1)	316(62.7)
Prematurity	119(23.6)	47(9.3)	338(67.1)
Mother’s age	18(3.6)	40(7.9)	444(88.1)
Mother’s blood group	33(6.5)	106(21.0)	362(71.8)
Eating certain foods eg palm oil	201(39.9)	5(1.0)	298(59.1)
Naphthalene balls (camphor)	12(2.4)	19(3.8)	473(93.8)
Spiritual forces	57(11.3)	33(6.5)	414(82.1)
**Part of the body where the first sign of NNJ can be seen**			
Eyes	346(68.7)	-	158(31.3)
Face	39 (7.7)	19(3.8)	445(88.5)
Body/Skin	57(11.3)	18(3.6)	429(85.1)
Palm and sole of feet	26(5.2)	19(3.8)	457(90.7)
**Signs of serious illness in a jaundiced newborn (according to mothers)** *			
Yellowness palm and sole of feet	186(36.9)	**-**	318(63.1)
Crying a lot	227(45.0)	3(0.6)	274(54.4)
Not feeding well	271(53.8)	3(0.6)	230(45.6)
Sleeping more than before	12(2.4)	46(9.1)	446(88.5)
Abnormal movements	26(5.2)	2(0.4)	474(94.0)
Convulsion/Seizure	77(15.3)	6(1.2)	420(83.3)

* multiple responses allowed.

### Knowledge about prevention and long-term effects

For the prevention of NNJ, 348 (69.0%), 210 (41.7%) and 136(27.0%) participants described antenatal clinic attendance, taking vitamins during pregnancy and delivery at a health facility respectively as the commonest activities for preventing NNJ. While 81(16.1%) mothers described knowledge of their blood group as a preventive measure, only 12(2.4%) knew that avoidance of naphthalene balls could prevent severe NNJ in the context of Ghana. The commonest long-term effect stated was death 348(61.9%), but knowledge about other long-term effects was low, specifically 167(33.1%) for delayed development, 79(15.7%) for epilepsy, 54(10.7%) for physical deformity, and 47(9.3%) for deafness (not included in tables).

### Attitudes and perceptions towards NNJ

Whilst 467 (92.7%) participants recommended that a mother should take their jaundiced baby to a health facility, only 358(71%) said they will go to a health facility and 68(13.5%) will inform the local midwife. Most mothers, 367(72.8%), reported that a mother should seek healthcare at the time NNJ is noticed. The commonest reasons given for seeking healthcare were treatment and prevention of death. Although 396(78.6%) of the women knew that NNJ could be treated, most did not know how NNJ is treated in the health facility (Table 3).

**Table 3:** Attitudes and perceptions of mothers towards NNJ.

Characteristic	Yes [frequency (%)]	No [frequency (%)]	Don’t know [frequency (%)]
**What should a mother do when her new-born becomes jaundiced?**			
Do nothing	3(0.6)	81(16.1)	420(83.3)
Give glucose water	26(5.2)	33(6.5)	445(88.3)
Put baby in the sun	47(9.3)	44(8.7)	413(81.9)
Go to clinic/hospital	358(71.0)	7(1.4)	139(27.6)
Tell the midwife	68(13.5)	12(2.4)	424(84.1)
Tell your pastor /Pray about it	12(2.4)	47(9.3)	445(88.3)
Use herbs	17(3.4)	46(9.1)	440(87.3)
**Should babies with jaundice be taken to a health facility for assessment?**	467(92.7)	12(2.4)	25(5.0)
**When should the mother seek healthcare**			
At the time she noticed jaundice	367(72.8)	11(2.2)	126(25.0)
If the baby is not feeding well	54(10.7)	40(7.9)	406(80.6)
If the baby is getting very yellow	41(8.1)	45(8.9)	414(82.1)
If the baby is crying abnormally	51(10.1)	44(8.7)	403(80.0)
**How do health workers check that jaundice is mild or severe** *			
Looking at the baby	124 (24.6)	24(4.8)	354(70.2)
Examining the baby very closely	80(15.9)	12(2.4)	411(81.5)
Doing blood tests	322(63.9)	8(1.6)	174(34.5)
**Can jaundice be treated?**	396(78.6)	4(0.8)	102(20.2)
**Mother’s perception of how jaundice is treated at the health facility** *			
Putting baby under special light	123(24.4)	8(1.6)	371(73.6)
Giving baby drip	67(13.3)	10(2.0)	424(84.1)
Giving baby injections	86(17.1)	10(2.0)	405(80.4)
Using a special blanket with light	-	6(1.2)	494(98.0)
Exchanging baby’s blood	2(0.4)	9(1.8)	489(97.0)

* multiple responses allowed.

None of the women knew what G6PD was. Though 12 out of the 14 who claimed they had been told their G6PD status said it was either ‘normal’ or ‘negative’, none of them understood what the results meant or how it was related to NNJ. Of the 15(3%) mothers who had their own child diagnosed with NNJ cost (8 out of 15) was the most perceived barrier to seeking healthcare. Other barriers highlighted by the mothers included the use of herbal medicines, being advised to expose the baby to sunlight, distance to the health facility and cultural beliefs.

### Selected characteristics of mothers and their knowledge, attitude, and perceptions

There were significant associations (p-value for the chi-square test of association ≤ 0.05) between maternal age and general understanding and knowledge about NNJ (Table 4). Mothers’ age, level of education and having a jaundiced child were significantly associated with adequate understanding and knowledge of the signs of severe disease and complications of NNJ (Table 5) and their attitude and perceptions about seeking healthcare and treatment of NNJ (Table 6). Most mothers who had prior experience of having a jaundiced newborn who was treated in hospital lacked knowledge about the causes, prevention, and complications of NNJ.

**Table 4:** Association between selected characteristics of mothers and knowledge of neonatal jaundice.

Characteristics	Understanding of neonatal jaundice		p-value	General knowledge about neonatal jaundice		p-value	Knowledge of the causes of neonatal jaundice		p-value
	adequate	inadequate		adequate	inadequate		adequate	inadequate	
**Age at last birthday (years)**									
≤ 19	20 (5.7)	118 (11.8)	0.03*	18 (5.2)	20 (12.9)	0.01*	3 (5.8)	35 (7.8)	0.55
20–29	167 (48.0)	80 (52.6)		169 (48.7)	78 (50.3)		30(57.7)	21748.2)	
30–39	141 (40.5)	50 (32.9)		140 (40.3)	53 (34.2)		16(30.8)	177 (39.3)	
40–49	20 (5.7)	4 (2.6)		20 (5.8)	4 (2.6)		3 (5.8)	21 (4.7)	
**Highest level of education**									
No formal or less than primary school completed	15 (4.3)	7 (4.6)	0.05*	17 (4.9)	5 (3,2)	0.16	3 (5.9)	19 (4.2)	0.87
Primary school completed	47 (13.5)	33 (21.9)		48 (13.9)	34 (21.9)		7 (13.7)	75 (16.7)	
Junior high school completed	151 (43.4)	55 (36.4)		141 (40.8)	65 (41.9)		20 (39.2)	186 (41.3)	
Senior high school/ vocational school completed	87 (25.0)	44 (29.1)		96 (27.7)	35 (22.6)		13 (25.5)	118 (26.2)	
College/University completed	48 (13.8)	12 (7.9)		44 (12.7)	16 (10.3)		8 (15.7)	52 (11.6)	
**Having a jaundiced child**									
Yes	14 (4.0)	1 (0.7)	<0.01*	11 (3.2)	4 (2.6)	0.37	1 (2.0)	14 (3.1)	0.43
No	392 (94.8)	127 (84.1)		317 (92.2)	140 (89.7)		49 (96.1)	408 (90.9)	
Don’t know	4 (1.2)	4 (15.2)		16 (4.7)	12 (7.7)		1 (2.0)	27 (6.0)	

* P-value for Pearson’s chi-square significant at 0.05

**Table 5:** Association between selected characteristics of mothers and knowledge of signs, prevention, and complications of severe neonatal jaundice.

	Knowledge of the signs of severe disease		p-value	Knowledge of prevention of severe disease		p-value	Knowledge of complications of NNJ		p-value
	adequate	inadequate		adequate	inadequate		adequate	inadequate	
**Age at last birthday (years)**									
≤ 19	24 (7.6)	14 (7.5)	0.97	9 (8.1)	29 (7.4)	0.25	17 (8.5)	21 (7.0)	0.05*
20–29	155 (49.1)	92 (49.5)		55 (49.5)	192 (49.1)		106 (53.3)	14 (46.4)	
30–39	123 (38.9)	70 (37.6)		38 (34.2)	155 (39.6)		63 (31.7)	130 (43.0)	
40–49	14 (4.4)	10 (5.4)		9 (8.1)	15 (3.8)		13 (6.5)	11 (3.6)	
**Highest level of education**									
No formal or less than primary school completed	14 (4.4)	8 (4.3)	0.46	4 (3.6)	18 (4.6)	0.98	10 (5.0)	12 (4.0)	0.06
Primary school completed	46 (14.6)	36 (19.5)		17 (15.3)	65 (16.7)		21 (10.6)	61 (20.3)	
Junior high school completed	131 (41.5)	75 (40.5)		47 (42.3)	159 (40.8)		90 (45.2)	116 (38.5)	
Senior high school/ vocational school completed	82 (25.9)	49 (26.5)		29 (26.1)	102 (26.2)		51 (25.6)	79 (26.2)	
College/University completed	43 (13.6)	17 (9.2)		14 (12.6)	46 (11.8)		27 (13.6)	33 (11.0)	
**Having a jaundiced child**									
Yes	11 (3.5)	4 (2.2)	<0.01	3 (2.7)	12 (3.1)	0.92	8 (4.1)	6 (2.0)	<0.01*
No	298 (94.9)	159 (85.5)		101 (91.0)	356 (91.5)		185 (40.5)	272 (59.5)	
Don’t know	5 (1.6)	23 (12.4)		7 (6.3)	21 (5.4)		3 (1.5)	25 (8.3)	

* P-value for Pearson’s chi-square significant at 0.05

**Table 6:** Association between selected characteristics of mothers and attitude/perceptions towards neonatal jaundice.

	What should a mother do when her new-born becomes jaundiced?		p-value	When the mother should seek healthcare		p-value	Can NNJ be treated?		p-value
	Appropriate	inappropriate		appropriate	inappropriate		appropriate	inappropriate	
**Age at last birthday (years)**									
≤ 19	25 (7.6)	13 (7.6)	0.19	33 (8.3)	5 (4.9)	0.45	20 (5.1)	18 (17.1)	<0.01*
20–29	168 (50.9)	79 (45.9)		194 (48.7)	52 (50.5)		199 (50.4)	47 (44.8)	
30–39	126 (38.2)	67 (39.0)		150 (37.7)	43 (41.7)		152 (38.5)	40 (38.1)	
40–49	11 (3.3)	13 (7.6)		21 (5.3)	3 (2.9)		24 (6.1)	0 (0.0)	
**Highest level of education**									
No formal or less than primary school completed	13 (3.9)	9 (5.3)	<0.01*	16 (4.0)	6 (5.9)	<0.01*	15 (3.8)	7 (6.7)	<0.01*
Primary school completed	46 (13.9)	36 (21.1)		53 (13.3)	29 (28.4)		52 (13.2)	29 (27.9)	
Junior high school completed	140 (42.4)	66 (38.6)		173 (43.5)	32 (31.4)		164 (41.5)	41 (39.4)	
Senior high school/ vocational school completed	81 (24.5)	50 (29.2)		103 (25.9)	28 (27.5)		110 (27.8)	21 (20.2)	
College/University completed	50 (15.2)	10 (5.8)		53 (13.3)	7 (6.9)		54 (13.7)	6 (5.8)	
**Having a jaundiced child**									
Yes	11 (3.3)	4 (2.3)	<0.01*	14 (3.5)	1 (1.0)	<0.01*	15 (3.8)	0 (0.0)	0.04*
No	309 (93.9)	148 (86.5)		373 (94.4)	83 (79.8)		361 (91.6)	95 (91.3)	
Don’t know	9 (2.7)	19 (11.1)		8 (2.0)	20 (19.2)		18 (4.6)	9 (8.7)	

* P-value for Pearson’s chi-square significant at 0.05

### Differences between health facilities

In general, there were significant differences between facilities with respect to knowledge about causes, signs of severe disease, prevention, complications, attitudes, and perceptions about NNJ. As shown in Table 7, significantly higher proportions of mothers accessing services at the Mamprobi Polyclinic in an urban poor setting were likely to have adequate knowledge of the causes, signs of severe disease, complications, attitudes, and perceptions about NNJ compared to Greater Accra Regional Hospital and Holy Family Hospitals (p-value < 0.05). There were no significant differences between the mothers in rural and urban locations with respect to knowledge about causes, signs of severe disease, prevention, complications, attitudes, and perceptions about NNJ.

**Table 7:** Differences in knowledge, attitude, and perceptions of mothers towards neonatal jaundice by health facilities accessed.

	Adequacy/ appropriateness	Hospital Type and Location			p-value
		District level, Urban Poor	Regional referral, Urban	District level, Rural	
General knowledge about neonatal jaundice	adequate	131 (78.0)	108 (64.3)	108 (64.3)	0.007
Knowledge of the causes of neonatal jaundice	adequate	26 (15.5)	14 (8.3)	13 (7.7)	0.037
Knowledge of the signs of severe disease	adequate	132 (78.6)	88 (52.4)	98 (58.3)	0.007
Knowledge of prevention of severe disease	adequate	15 (8.9)	62 (36.9)	35 (20.8)	<0.001
Knowledge of complications of NNJ	adequate	96 (57.1)	46 (27.4)	57 (34.1)	<0.001
What should a mother do when her new-born becomes jaundiced?	appropriate	141 (83.9)	87 (51.8)	103 (61.3)	<0.001
When the mother should seek healthcare	appropriate	144 (85.7)	122 (73.1)	133 (79.2)	0.017
Can NNJ be treated?	appropriate	153 (91.1)	119 (71.3)	124 (74.3)	<0.001

## Discussion

This study revealed that most mothers had heard about NNJ, knew the condition was associated with yellowing of the eyes and that it may be harmful to babies, but did not know the causes or signs and symptoms of severe disease although they would seek orthodox healthcare for the jaundiced baby because they believe NNJ could be treated. None of the mothers had any understanding about G6PD and how it is related to NNJ. Cost was the most perceived and experienced barrier to accessing healthcare for the jaundiced newborn. Maternal age, educational level and having a previously diagnosed jaundiced child were significantly associated with general understanding and care seeking for NNJ. Women attending the health facility in an urban poor setting had significantly better knowledge, attitude and perception about NNJ than women in the rural and urban referral hospitals These findings highlight the gaps and opportunities in the health system and the urgent need to improve population health literacy on NNJ as a strategy for improving neonatal health outcomes [21].

Early detection and management of hyperbilirubinaemia beyond the threshold for postnatal age is key to reducing the burden of disease associated with NNJ. High level community awareness and effective screening methods at the point of care are critical to early detection. The high level of awareness in this study is consistent with studies conducted in other low- middle-income countries [17, 22, 23] where health workers were also the primary source of information on NNJ [23, 24]. Yellowing of the eyes was what most mothers understood as jaundice and the earliest sign of NNJ. This is similar to reports from mothers in Nepal [25] and Nigeria [14] but in contrast to findings in Egypt [15]. The level of melanin pigmentation in the newborn varies between races and ethnic groups and may affect the ability of mothers and health workers to assess yellowness in the skin even in the first week after birth [26]. Visual identification of NNJ on the skin is unreliable, especially in sub-Saharan African newborns; implementation of new technologies for screening newborns for jaundice [27, 28] should consider culturally acceptable practices in the identification of NNJ.

The low-level knowledge among mothers with respect to the causes, signs and symptoms of severe NNJ was not consistent with the relatively higher-level knowledge in other studies conducted in urban communities in Nigeria [14] and Ghana [19]. Nevertheless, similar causes of NNJ such as eating palm oil and low-level knowledge about blood group incompatibilities were reported. Higher levels of knowledge in urban communities have been described [15] and was confirmed for the urban poor population in this study, however, the similar levels of knowledge and perception among participants at the urban regional referral hospital and the rural hospital was unexpected.

Although the low-level knowledge of danger signs and long-term effects of NNJ was worrying, the findings indicate that majority of mothers perceived NNJ as a treatable condition (78.6%) and would advocate seeking care at a health facility (92.7%). This is encouraging and may imply that effective community-based screening could potentially reduce delays in care seeking and long-term morbidity associated with NNJ. As described by others, increasing maternal age, educational level, and having a previously diagnosed jaundiced child were significantly associated with adequate understanding of NNJ and care seeking [14, 17]. This finding provides evidence for identifying mothers who could become champions for NNJ prevention in communities.

None of the mothers had any understanding about G6PD despite laboratory tests for G6PD being part of free maternal care for all women attending antenatal clinics in Ghana [20]. The relevance of maternal G6PD test for the newborn was unknown to the mothers. Naphthalene balls are commonly used in Ghana [29] but only 2.4% of mothers reported it may cause severe NNJ. The lack of knowledge on G6PD is a major concern as an estimated 15–26% of the Ghanaian population have G6PD defect [30] and there is established evidence of its association with hyperbilirubinaemia [31] when infants with the defect are exposed to oxidants such as naphthalene balls [32]. Also, the low level of understanding about causes, prevention, and complications of NNJ among the 15 women who had prior hospitalization for a jaundiced infant is concerning. There is a need to improve population health literacy and improve the content of health education provided to mothers during antenatal and postnatal newborn care and hospitalization of newborns with NNJ.

The comparatively large sample, inclusion of women from rural and urban communities at different levels and periods of obstetric care, and assessment of multiple variables for knowledge, attitude and perceptions contribute to the strength of this study. The inherent limitations of using interviewer administered questionnaires [33] is acknowledged but majority of the study population did not have high levels of education required for self-administered questionnaires. There is no published standardized questionnaire for evaluating the variables in the population of interest examined in this study; we adapted the questionnaire from several published studies on NNJ and pretested it before implementation in the field [34]. The study was limited to 2 regions in the country hence the results of the study may not be generalizable to women in the entire country.

The findings of this study provide relevant information that new health technologies could leverage for future directions in screening and referral systems for NNJ. Although most mothers recommend early healthcare seeking for NNJ, they also reported that cost, distance to travel and cultural beliefs were barriers to seeking care and most did not know the signs of complicated NNJ. There is increasing ownership of smartphones and use of mobile health technologies in sub-Saharan Africa [35]. Understanding local health practices, including gaps and opportunities for improving care for infants with hyperbilirubinaemia, may enhance and sustain implementation of new technologies that address the barriers identified in this study.

## Conclusion

Effective implementation of new health technologies requires a good understanding of the target population and healthcare systems. In this study, there were significant gaps in knowledge of the real factors associated with poor health outcomes in NNJ despite the high level of awareness among this representative population of women of reproductive age in Ghana. The high number of women advocating for facility-based care for jaundiced infants indicate that effective screening strategies have the potential to reduce delays in seeking healthcare, but hospitals require adequate resources, especially phototherapy equipment, to meet the need. The low-level of knowledge about the link between NNJ and routine antenatal screening test, specifically blood group type and G6PD status needs to be addressed. Opportunities exist for implementing timely interventions to arrest NNJ progression to kernicterus and the long-term neurological impairment faced by survivors.
